# Supplementary material for: Evolutionary History With Chronic Malnutrition Enhances Pathogen Susceptibility at Older Ages
Source: Ecol Evol. 2025 Apr 3;15(4):e71070. doi: 10.1002/ece3.71070 (PMC11968410; doi:10.1002/ece3.71070)
Supplement: Supplementary file 2 — Data S2 [file ECE3-15-e71070-s002.docx]

**SUPPLEMENTARY INFORMATION**

**TABLES**

**Table S1**. Summary of a mixed effect Cox model, using selection regime and infection dose as fixed effects and vial identity nested within replicate populations as a random effect (Model: *Post-infection survival ~ Infection dose* ***×*** *Selection regime + 1|* *Replicate population/ Vial*). Statistically significant p-values are highlighted in bold.

|  |  |  | **Young** | | | | | | |  | | **Old** | | | | | | |  |
| --- | --- | --- | --- | --- | --- | --- | --- | --- | --- | --- | --- | --- | --- | --- | --- | --- | --- | --- | --- |
| Full model | ***Tested effect*** |  | *Chisq* | | *Df* | | *p value* | | |  | | *Chisq* | *Df* | | *p value* | | | |  |
|  | *Infection dose (ID)* |  | 395.456 | | 4 | | **<2.2e-16***** | | |  | | 405.748 | 4 | | **<2.2e-16***** | | | |  |
|  | *Selection Regime (SR)* |  | 15.640 | | 1 | | **7.662e-05***** | | |  | | 65.7112 | 1 | | **5.221e16***** | | |  |  |
|  | *ID* ***✕ SR*** |  | 31.344 | | 4 | | **2.605e-06***** | | |  | | 4.3667 | 4 | | 0.3586 | | | |  |
| Post hoc to compare regime across doses | *ID* |  | *Estimate* | *SE* | | | *Z ratio* | *p-value* | | |  | *Estimate* | | *SE* | | *Z ratio* | *p-value* | | |
|  | *0.05 OD* |  | -0.308 | | | 0.111 | -2.788 | | 0.14 | |  | -0.441 | | 0.131 | | -3.360 | **0.027** | |  |
|  | *0.1 OD* |  | 0.242 | | | 0.106 | 2.295 | | 0.392 | |  | -0.455 | | 0.122 | | -3.731 | **0.007** | |  |
|  | *0.5 OD* |  | 0.447 | | | 0.103 | 4.338 | | **0.001** | |  | -0.640 | | 0.113 | | -5.677 | **<0.001** | |  |
|  | *1 OD* |  | 0.409 | | | 0.104 | 3.948 | | **0.003** | |  | -0.405 | | 0.109 | | -3.725 | **0.008** | |  |

**Table S2**. Summary of a generalised linear mixed-effects model, with selection regime and infection dose as fixed effects and vial identity nested within the replicate populations as a random effect (Model: *Log average bacterial load~ Infection dose* ***×*** *Selection regime* ***+ 1|Replicate population/Vial****, family=negative binomial*). Statistically significant p-values have been highlighted in bold.

|  |  | |  | **Young** | | | | | | |  | | **Old** | | | | | | |  |
| --- | --- | --- | --- | --- | --- | --- | --- | --- | --- | --- | --- | --- | --- | --- | --- | --- | --- | --- | --- | --- |
| Full model | | ***Tested effects*** |  | *Chisq* | | *Df* | *p-value* | | | |  | | *Chisq* | *Df* | | *p-value* | | | |  |
|  |  | *Infection dose (ID)* |  | 3.548 | | 3 | 0.314 | | | |  | | 28.508 | 3 | | **2.842e-06***** | | | |  |
|  |  | *Selection Regime (SR)* |  | 0.258 | | 1 | 0.612 | | | |  | | 1.361 | 1 | | 0.24331 | | |  |  |
|  |  | *ID* ***✕ SR*** |  | 1.074 | | 3 | 0.783 | | | |  | | 6.596 | 3 | | 0.08595 | | | |  |
| Post hoc to compare regime across doses | | *ID* |  | *Estimate* | *SE* | | | *Z ratio* | *p-value* | | |  | *estimate* | | *SE* | | *Z ratio* | *p-value* | | |
|  |  | *0.05 OD* |  | -0.075 | 0.103 | | | -0.729 | | 0.996 | |  | 0.652 | | 0.237 | | 2.746 | 0.109 | | |
|  |  | *0.1 OD* |  | -0.108 | 0.143 | | | -0.759 | | 0.9950 | |  | 0.201 | | 0.258 | | 0.776 | 0.994 | | |
|  |  | *0.5 OD* |  | 0.014 | 0.152 | | | 0.092 | | 1.000 | |  | 0.008 | | 0.199 | | 0.039 | 1.000 | | |
|  |  | *1 OD* |  | 0.043 | 0.149 | | | 0.288 | | 1.000 | |  | -0.021 | | 0.194 | | -0.107 | 1.000 | | |

**Table S3**. Summary of the generalised linear model fitted separately for young and old flies, using log-transformed hazard function (HR) as a response variable, pseudo-log-transformed bacterial load (BL) as a covariate and selection regime (SR) as a fixed effect (model: *Log hazard ratio~ Log average bacterial load* **×** *Selection regime, family=negative binomial*). A significant interaction between bacterial load and selection regime indicates variation in infection tolerance. Statistically significant P-values have been highlighted in bold.

|  |  | **Young** | | |  | **Old** | | |
| --- | --- | --- | --- | --- | --- | --- | --- | --- |
| **Tested effect** |  | *Chisq* | *Df* | *p-value* |  | *Chisq* | *Df* | *p-value* |
| Log Avg bacterial load (BL) |  | 0.7710 | 1 | 0.37990 |  | 42.836 | 1 | **5.951e-11 ***** |
| Selection regime (SR) |  | 0.2294 | 1 | 0.63194 |  | 12.074 | 1 | **0.0005112 ***** |
| BL **✕ SR** |  | 6.3165 | 1 | **0.01196*** |  | 0.545 | 1 | 0.4602286 |


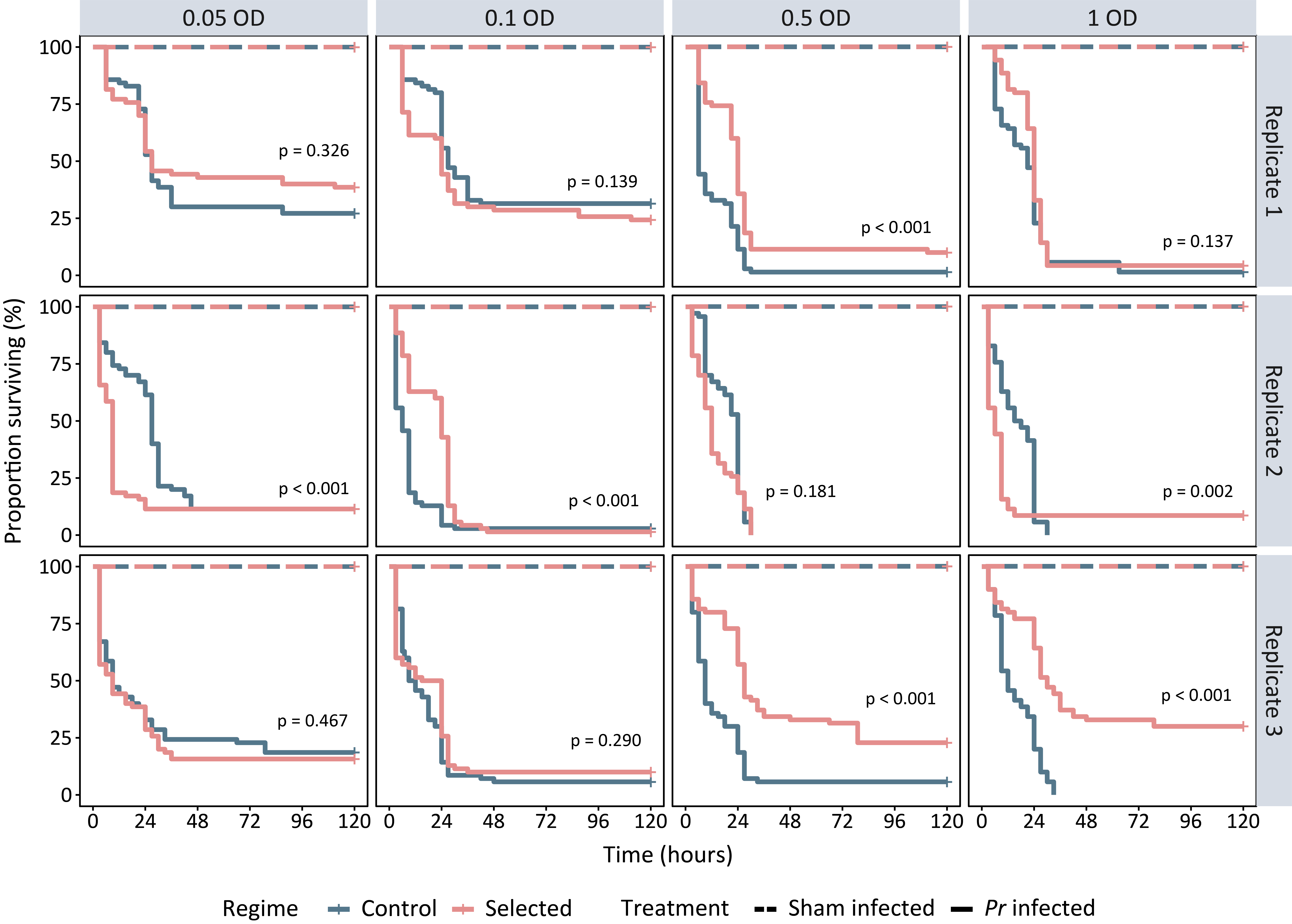
**Figure S1.** Post-infection survival data of replicate populations of selected flies and their control, 120 hours post *P. rettgeri* infection at a young age. The p values indicate the difference between control and selected regimes after *Pr* infection.


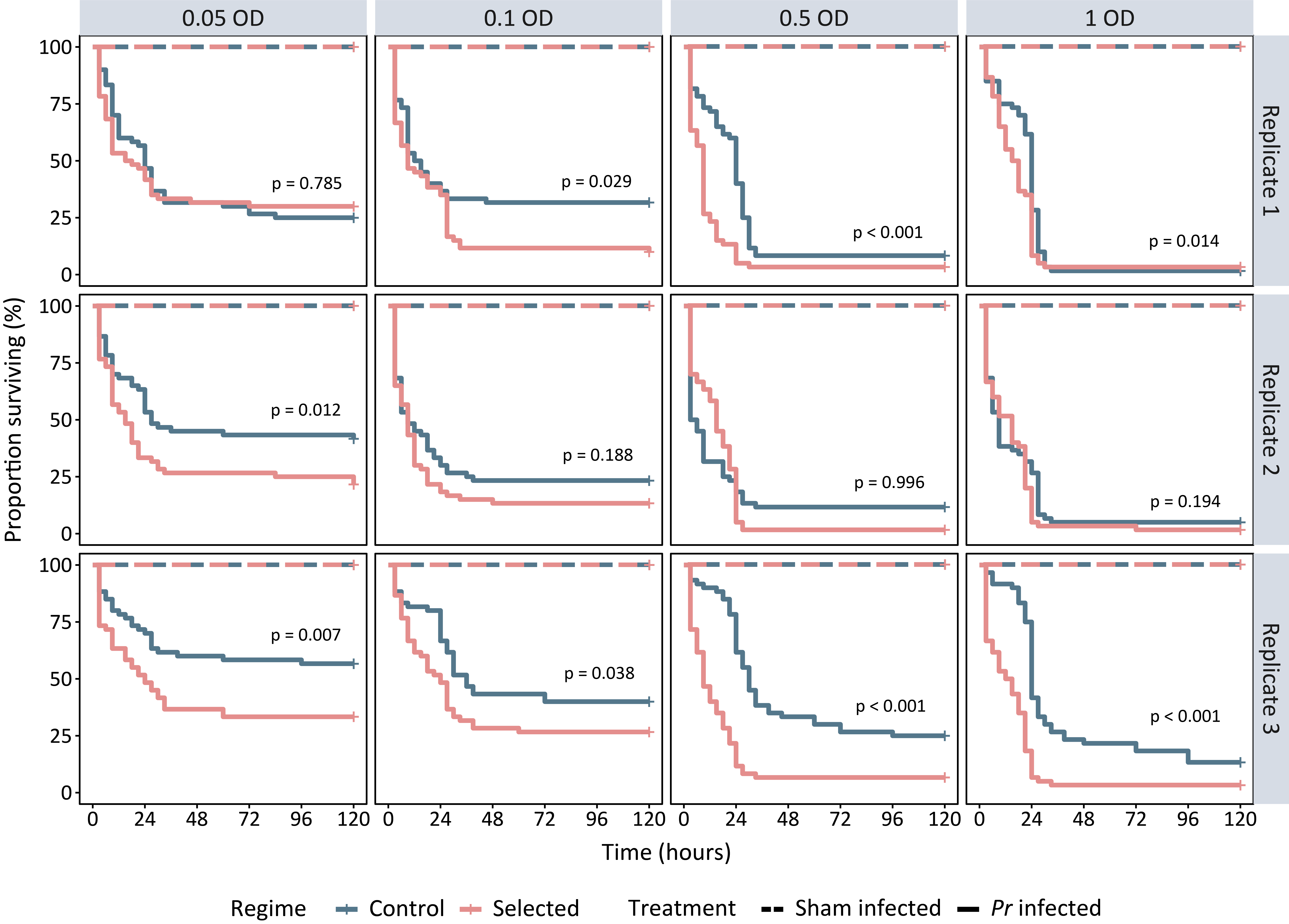
**Figure S2.** Post-infection survival data of replicate populations of selected flies and their control, 120 hours post *P. rettgeri* infection at old age. The p values indicate the difference between control and selected regimes after *Pr* infection.

**Table S4**. The effect of infection dose and selection regime on survival of individual replicate fly population 120 h post-infection with *Providencia rettgeri* at a young age. Values in bold are statistically significant.

|  |  |  | Tested effect | | | Chisq | Df | | p-value |
| --- | --- | --- | --- | --- | --- | --- | --- | --- | --- |
| **Young** | *Replicate set 1* | *Full model* | *Dose*  *Regime*  *Dose* ***✕ Regime*** | | | 199.703 | 4 | | **< 2.2e-16 ***** |
|  |  |  |  |  |  | 10.639 | 1 | | **0.0011072**** |
|  |  |  |  |  |  | 21.895 | 4 | | **0.0002103***** |
|  |  | Dose specific comparisons across regimes | *Dose* | *loglik* | | *Chisq* | *Df* | | *p-value* |
|  |  |  | *0.05 OD* | -421.78 | | 0.9658 | 1 | | 0.3257 |
|  |  |  | *0.1 OD* | -447.49 | | 2.1946 | 1 | | 0.1385 |
|  |  |  | *0.5 OD* | -532.66 | | 23.905 | 1 | | **1.012e-06 ***** |
|  |  |  | *1 OD* | -550.93 | | 2.2173 | 1 | | 0.1365 |
|  | *Replicate set 2* | *Full model* | *Dose* |  | | 168.964 | 4 | | **<2.2e-16***** |
|  |  |  | *Regime* |  | | 1.237 | 1 | | 0.266 |
|  |  |  | *Dose* ***✕ Regime*** | |  | 58.326 | 4 | | **6.518e-12***** |
|  |  | Dose specific comparisons across regimes | *Dose* | *loglik* | | *Chisq* | *Df* | | *p-value* |
|  |  |  | *0.05 OD* | -517.48 | | 14.129 | 1 | | **0.0001707 ***** |
|  |  |  | *0.1 OD* | -541.47 | | 23.907 | 1 | | **1.011e-06 ***** |
|  |  |  | *0.5 OD* | -554.32 | | 1.793 | 1 | | 0.1806 |
|  |  |  | *1 OD* | -543.72 | | 9.839 | 1 | | **0.001708 **** |
|  | *Replicate set 3* | *Full model* | *Dose* |  | | 127.469 | 4 | **< 2.2e-16 ***** | |
|  |  |  | *Regime* |  | | 25.471 | 1 | **4.491e-07 ***** | |
|  |  |  | *Dose* ***✕ Regime*** | |  | 35.697 | 4 | **3.341e-06 ***** | |
|  |  | Dose specific comparisons across regimes | *Dose* | *loglik* | | *Chisq* | *Df* | | *p-value* |
|  |  |  | *0.05 OD* | -500.17 | | 0.5282 | 1 | | 0.4674 |
|  |  |  | *0.1 OD* | -537.16 | | 1.1202 | 1 | | 0.2899 |
|  |  |  | *0.5 OD* | -500.24 | | 25.291 | 1 | | **4.931e-07 ***** |
|  |  |  | *1 OD* | -487.50 | | 44.678 | 1 | | **2.322e-11 ***** |

**Table S5**. The effect of infection dose and selection regime on survival of individual replicate fly population 120 post-infection with *Providencia rettgeri* at an old age. Values in bold are statistically significant.

|  |  |  | Tested effect | | | Chisq | Df | | p-value |
| --- | --- | --- | --- | --- | --- | --- | --- | --- | --- |
| **Old** | *Replicate set 1* | *Full model* | *Dose*  *Regime*  *Dose* ***✕ Regime*** | | | 134.354 | 4 | | **< 2.2e-16 ***** |
|  |  |  |  |  |  | 18.979 | 1 | | **1.322e-05***** |
|  |  |  |  |  |  | 10.330 | 4 | | **0.03522*** |
|  |  | Dose specific comparisons across regimes | *Dose* | *loglik* | | *Chisq* | *Df* | | *p-value* |
|  |  |  | *0.05 OD* | -372.72 | | 0.0743 | 1 | | 0.7852 |
|  |  |  | *0.1 OD* | -397.42 | | 4.779 | 1 | | **0.02881 *** |
|  |  |  | *0.5 OD* | -438.24 | | 22.092 | 1 | | **2.599e-06 ***** |
|  |  |  | *1 OD* | -453.00 | | 6.0421 | 1 | | **0.01397 *** |
|  | *Replicate set 2* | *Full model* | *Dose* |  | | 137.049 | 4 | | **<2.2e-16***** |
|  |  |  | *Regime* |  | | 5.081 | 1 | | **0.02419*** |
|  |  |  | *Dose* ***✕ Regime*** | |  | 5.048 | 4 | | 0.28242 |
|  |  | Dose specific comparisons across regimes | *Dose* | *loglik* | | *Chisq* | *Df* | | *p-value* |
|  |  |  | *0.05 OD* | -351.72 | | 6.2551 | 1 | | **0.01238 *** |
|  |  |  | *0.1 OD* | -408.47 | | 1.7326 | 1 | | 0.1881 |
|  |  |  | *0.5 OD* | -447.21 | | 0 | 1 | | 0.996 |
|  |  |  | *1 OD* | -453.79 | | 1.6845 | 1 | | 0.1943 |
|  | *Replicate set 3* | *Full model* | *Dose* |  | | 143.624 | 4 | | **< 2.2e-16 ***** |
|  |  |  | *Regime* |  | | 60.324 | 1 | **8.045e-15 ***** | |
|  |  |  | *Dose* ***✕ Regime*** | |  | 6.552 | 4 | | 0.1616 |
|  |  | Dose specific comparisons across regimes | *Dose* | *loglik* | | *Chisq* | *Df* | | *p-value* |
|  |  |  | *0.05 OD* | -293.49 | | 7.1567 | 1 | | **0.007469 **** |
|  |  |  | *0.1 OD* | -345.34 | | 4.3112 | 1 | | **0.03786 *** |
|  |  |  | *0.5 OD* | -402.18 | | 32.582 | 1 | | **1.143e-08 ***** |
|  |  |  | *1 OD* | -426.61 | | 32.19 | 1 | | **1.398e-08 ***** |
